# Pathways linking microbiota-gut-brain axis with neuroinflammatory mechanisms in Alzheimer’s pathophysiology

**DOI:** 10.20517/mrr.2023.39

**Published:** 2023-12-06

**Authors:** Nathaniel Hochuli, Saurabh Kadyan, Gwoncheol Park, Cole Patoine, Ravinder Nagpal

**Affiliations:** Department of Health, Nutrition, and Food Sciences, College of Education, Health, and Human Sciences, Florida State University, Tallahassee, FL 32306, USA.

**Keywords:** Neurodegeneration, neuroinflammation, gut dysbiosis, inflammatory cytokines, microbiota-gut-brain axis, microglia activation

## Abstract

Disturbances in the local and peripheral immune systems are closely linked to a wide range of diseases. In the context of neurodegenerative disorders such as Alzheimer’s disease (AD), inflammation plays a crucial role, often appearing as a common manifestation despite the variability in the occurrence of other pathophysiological hallmarks. Thus, combating neuroinflammation holds promise in treating complex pathophysiological diseases like AD. Growing evidence suggests the gut microbiome’s crucial role in shaping the pathogenesis of AD by influencing inflammatory mediators. Gut dysbiosis can potentially activate neuroinflammatory pathways through bidirectional signaling of the gut-brain axis; however, the precise mechanisms of this complex interweaved network remain largely unclear. In these milieus, this review attempts to summarize the contributing role of gut microbiome-mediated neuroinflammatory signals in AD pathophysiology, while also pondering potential mechanisms through which commensal and pathogenic gut microbes affect neuroinflammation. While certain taxa such as *Roseburia* and *Escherichia* have been strongly correlated with AD, other clades such as *Bacteroides* and *Faecalibacterium* exhibit variations at the species and strain levels. In order to disentangle the inflammatory aspects of neurodegeneration attributed to the gut microbiome, it is imperative that future mechanistic studies investigate the species/strain-level dependency of commensals, opportunistic, and pathogenic gut microbes that consistently show correlations with AD patients across multiple associative studies.

## INTRODUCTION

The gut microbiome consists of trillions of fungal, archaeal, and bacterial species that feed off their hosts and each other to form a complex, interweaving web of host-modulating interactions^[1]^. The gut microbiome, in its homeostatic form, can confer many health benefits to the host via its regulation of digestive metabolism, immune system, and endocrine system towards the sustenance of optimal local (gut) and systemic (other bodily organs) functions. Its dysfunction has been implicated in the dysregulation of these functions, thereby contributing to the development of various chronic non-communicable disease states^[2]^. Thus far, research has demonstrated the causal versus merely correlational relationships between a dysbiotic gut microbiome and many lifestyle diseases, including cardiovascular diseases, diabetes, and inflammatory bowel diseases (IBD). Moreover, there is evidence suggesting that these disease states can potentially be improved by modulating the microbiome through environmental factors such as diet, exercise, and supplementation^[3]^. These diseases have further been implicated in the development of chronic neurodegenerative conditions; however, the specific mechanisms by which the gut microbiome exacerbates the pathophysiological hallmarks of these diseases and related neurodegenerative diseases remain enigmatic^[4]^. Emerging research proposed neuroinflammation as a possible factor bridging neurodegeneration and the gut microbiome via complex bidirectional interactions between gut microbes, the peripheral immune system, and neurodegeneration.

Neuroinflammation is a pathophysiological marker that is a commonly presenting sign despite the high variability of other pathophysiological markers in Alzheimer’s disease (AD) patients. A study by Leng *et al.* showed positive associations between neurodegeneration and neuroinflammation irrespective of β-amyloid (Aβ) deposition, a typical hallmark of AD, thus providing insight into the prominent role of neuroinflammation in triggering the pathogenesis of AD^[5]^. Moreover, inhibition of genes related to innate immunity, such as CD33, has shown a decreased risk for AD within Aβ-related pathologies^[6]^. Although neuroinflammation typically manifests as small, subclinical events, prolonged exposure to self-propagating positive feedback mechanisms can eventually lead to its overstimulation and subsequent neurodegeneration^[7]^. The involvement of neuroinflammation in many AD-related dysregulated processes has prompted many researchers to seek its inhibition as a preventative treatment pathway^[8]^. Given the extensive involvement of the gut microbiome in regulating a host’s immune responses, microbiome-targeted modulation has the potential to mitigate neuroinflammation-induced neurodegeneration over time^[9]^. However, achieving this goal necessitates a comprehensive understanding of AD pathophysiology and characterization of the proinflammatory and anti-inflammatory phenotypes within the gut-microbiota-immune axis that contribute to the propagation or attenuation of neuroinflammation. With this in mind, this review summarizes the current research and highlights the correlative evidence and causative mechanisms by which the gut microbiome may influence peripheral inflammation and neuroinflammation in a host with AD.

## NEUROINFLAMMATION - A TRIGGER FOR AD

AD is a chronic neurodegenerative disease responsible for around 60%-70% of the 55 million dementia cases worldwide^[10]^. In the ever-changing landscape of modern medicine, populations are experiencing increased average ages paralleled by an increase in AD patients, which is projected to exceed 139 million people by 2050^[11]^. Current treatment strategies merely act to prohibit symptomology rather than directly prevent or decrease the progression of the disease itself^[8]^.

While treatments for the disease have yet to be discovered, the field of AD research is rapidly evolving, and numerous therapeutic tracks are being explored. AD pathophysiology is primarily marked by the extracellular presence of Aβ plaques and intraneuronal neurofibrillary tangles (NFTs)^[12]^. The former is derived from amyloid precursor protein (APP) - a protein whose native function involves the positive regulation of memory and learning. Improper cleavage of APP to Aβ_42_ causes the oligomerization and misfolding of the protein into Aβ plaques, resulting in an impaired cognitive function^[13]^. NFTs are a product of hyperphosphorylated tau (pTau) proteins, and their intraneuronal accumulation directly leads to neuron death^[14]^. The precise mechanisms by which Aβ and pTau cause AD, however, remain unclear. The current accepted Amyloid Hypothesis posits that the accumulation of Aβ_42_ in cerebrospinal fluid (CSF) causes intracellular NFT formation, ultimately resulting in neuronal death^[12]^. This hypothesis has been recently called into question due to the ineffective nature of Aβ-reducing drugs and a lack of overlap in the primary physical location of Aβ and pTau in AD patients. This is especially true for late-onset sporadic AD, which composes a majority of AD cases^[12]^. As Aβ and pTau pathophysiology have both proven difficult as treatment targets, other associated deregulatory mechanisms, such as neuroinflammation, are also being assessed. Since the highly modulable gut microbiome is implicated in neuroinflammatory processes, this mechanism of neurodegeneration poses an interesting target for its amelioration.

**Figure 1:**
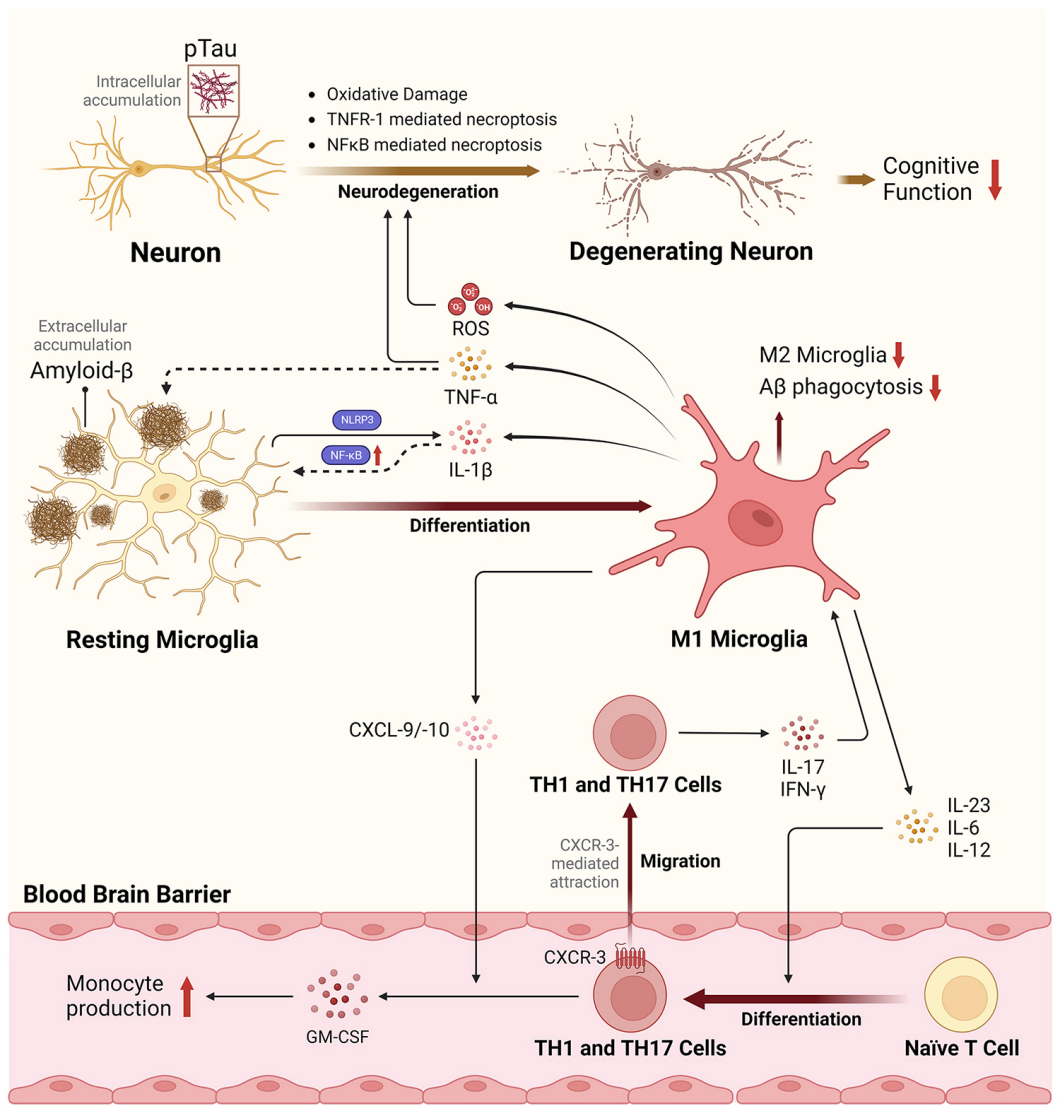
Mechanisms and role of Microglial-induced neuroinflammation in triggering AD pathophysiology. The neuroinflammatory pathway begins with the engulfment of amyloid-β peptide by resting microglia. This process activates the NLRP3 inflammasome and NFκB pathways, triggering the maturation of microglia into a proinflammatory M1 phenotype, which in turn stimulates the differentiation of naïve T-cells into T_H_1 and T_H_17 cells and facilitates their recruitment across the blood-brain barrier. This cascade leads to increased proinflammatory T-cells, cytokines, chemokines, and ROS within the brain environment, thereby driving the maturation and proliferation of M1 microglia, exacerbating oxidative damage, TFNR-1- and NFκB-mediated necroptosis, and resulting in the intracellular accumulation of pTau proteins. Ultimately, this cascade of events leads to neuronal death and cognitive decline. AD: Alzheimer’s disease; CXCR: C-X-C chemokine receptors; GM-CSF: granulocyte-macrophage colony-stimulating factor; IL: interleukin; NFκB: nuclear factor kappa B; NLRP3: NOD-like receptor family, pyrin domain-containing protein 3; pTau: phosphorylated tau protein; ROS: reactive oxygen species; TNF-α: tumor necrosis factor alpha; TNFR-1: tumor necrosis factor receptor 1.

The links between AD pathophysiology and neuroinflammation seem to be primarily mediated by the innate immune system and the activation of microglia - a brain-specific monocyte^[15]^. Microglia generally exhibit either a proinflammatory (M1) or anti-inflammatory phenotype (M2), each of which is positively or negatively correlated with AD, respectively^[16,17]^. The microglial-related neuroinflammatory pathways associated with AD pathogenesis are depicted in Figure 1. Microglial phagocytosis of Aβ has been shown to trigger the NLRP3 inflammasome for the production of interleukin-1β (IL-1β) - a cytokine that feeds back to stimulate microglial activation and upregulation of Nuclear Factor κB (NFκB)^[18,19]^. NFκB triggers microglial maturation to the highly proinflammatory M1 phenotype to produce reactive oxygen species (ROS), complement proteins, proinflammatory cytokines [IL-1β, IL-6, tumor necrosis factor-α (TNF-α), IL-12, and IL-23], and chemokines (CXCL-1/2, CXCL-10)^[16,19,20]^. Many of these factors influence M1 microglial maturation and T-cell differentiation, and their expression levels are significantly upregulated in AD patients^[16,19,20]^. TNF-α produced by M1 microglia can induce further neuroinflammation and neurodegeneration via TNFR1-mediated necroptosis - a form of programmed cell death resulting in further proinflammatory effects^[21]^. Moreover, the overexpression of these M1 microglial cytokines can suspend Aβ phagocytosis^[22]^, a job mediated primarily by M2 macrophages^[23]^. The presence of pTau itself in the cultured hippocampal neurons also caused increased IL-6, TNF-α, CXCL-2, and CXCL-10, as well as neuronal degeneration via NFκB-mediated necroptosis^[21]^. Other functions of M1 microglia, such as ROS production, may result in oxidative damage to proteins and lipids in neurons, thus further potentiating neurodegeneration, as reviewed by Ganguly *et al.*^[24]^. As a whole, it is generally the chronic, rather than acute, upregulation of these functions that seems to be responsible for neurodegenerative diseases such as AD^[7]^.

T-cells are also highly relevant in the pathophysiology of AD, and their differentiation can be stimulated via M1 microglia production of IL-12 and IL-23^[25]^. Naïve T-cells are produced in the thymus and alone do not cross the blood-brain barrier (BBB)^[26]^. A study by Browne *et al.*^[25]^, however, showed a significant fraction of activated interferon (IFN)-γ-producing (T_H_1) and IL-17-producing (T_H_17) cell recruitment across the BBB paralleling deposition of Aβ. The recruitment of fully differentiated T-cells across the BBB is not yet fully understood; however, it is hypothesized that the expression of CXCR-3 on T_H_1 and T_H_17 cells, coupled with the upregulation of its ligands CXCL-9 and CXCL-10 triggered by IFN-γ and pTau in AD, may contribute to this mechanism^[27]^. IL-17 produced by recruited T_H_17 cells has been observed following Aβ production in mouse model hippocampus and cortex regions^[28]^. Paralleled increases in CXCL-10, TNF-α, and cognitive impairment as a function of increased IL-17 have also been observed^[28]^. Moreover, blunted responses to T_H_17 cells via administration of IL-17 neutralizing antibodies showed a decrease in granulocyte-macrophage colony-stimulating factor (GM-CSF), thereby decreasing microglial proliferation and activation in mouse models^[28,29]^. The action of T_H_1 and T_H_17 cells in AD can thus be summarized by their production of proinflammatory cytokines and further stimulation and activation of microglia towards an M1 phenotype.

Overall, attempts at immunomodulation have proven somewhat successful in AD prevention studies. For instance, non-steroidal anti-inflammatory drugs (NSAIDs) have shown promise in the treatment of mild forms of AD; however, their implementation must occur over at least a 2-year period and beneficial effects are less obvious in older adult patients^[15]^. Moreover, long-term NSAID usage may potentially induce damage to intestinal epithelial cells, leading to increased gut permeability, which, in turn, results in leaky gut syndrome and eventually counteracts desired effects by promoting peripheral inflammation^[30]^. Considering that the gut microbiome offers an alternative, long-term pathway through which peripheral inflammation can be modulated, its role in AD is further investigated in subsequent sections to understand its impact on neuroinflammation.

### Novel mechanisms linking microglia and neuroinflammation: findings from single-cell RNA sequence research

**Figure 2:**
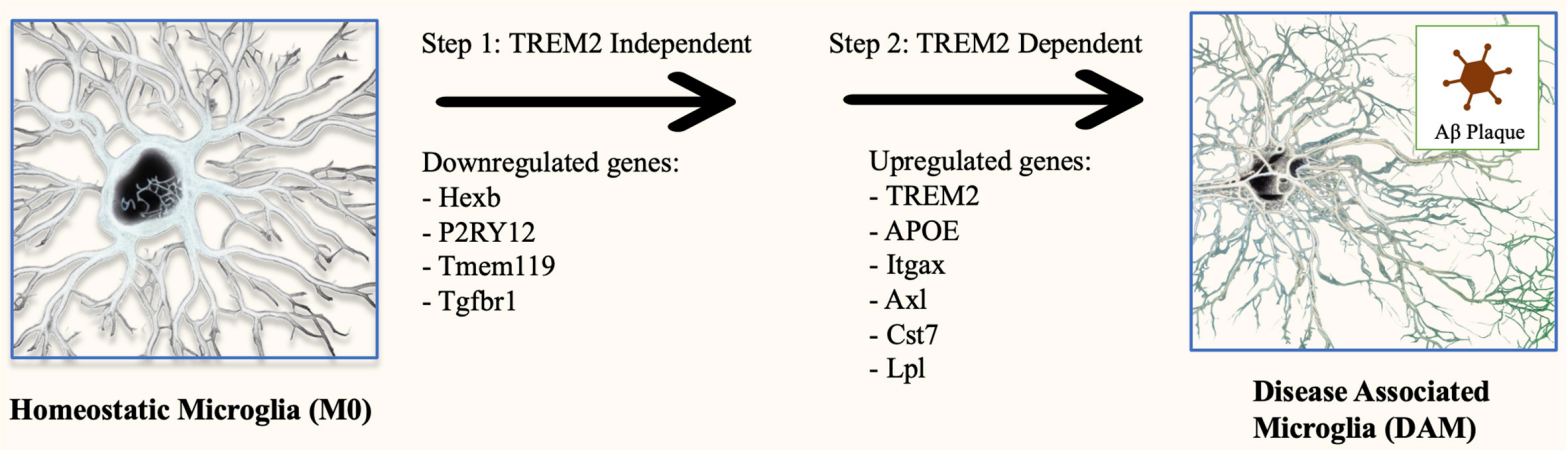
Two-step activation of DAM. Homeostatic microglia are differentiated into the DAM phenotype via a sequential two-step process. It begins with TREM2 independent change to an intermediate state. The cause of this initial change is not well known, but it is marked by the downregulation of homeostatic genes such as purinergic receptor P2RY12 and transmembrane protein 119 (Tmem119). This intermediate state is then activated to the DAM phenotype through a TREM2 signal-dependent second step involving the upregulation of many genes, such as those involved in phagocytosis (TREM2, Axl), lipid metabolism (APOE, Lpl), and inflammation (IL-1β), among others. APOE: Apolipoprotein E; Axl: Axl receptor tyrosine kinase; Cst7: cystatin F; Hexb: hexosaminidase subunit beta; Itgax: integrin subunit alpha X (also known as CD11c); Lpl: lipoprotein lipase; P2RY12: purinergic receptor P2Y12; Tgfbr1: transforming growth factor beta receptor 1; Tmem119: transmembrane protein 119; TREM2: triggering receptor expressed on myeloid cells 2.

Single-cell RNA sequencing (scRNA-seq) of microglia in recent research highlights the richness of human microglia heterogeneity and expands beyond the conventional understanding of M1 and M2 phenotypes^[31]^. By profiling individual cells at the transcriptomic level, specific cell-cell functional differences can be determined^[32]^. Most notably, a recent study conducted by Keren-Shaul *et al.* (2017) utilized scRNA-seq in a mouse model to identify a new subset of microglia denoted as disease-associated microglia (DAM) that localized around A plaques and exhibited potentially neuroprotective properties in the context of AD^[33]^. DAM commonly exhibit elevated lipid metabolism pathways and phagocytic-related genes. Their differentiation is thought to occur via a two-step triggering receptor expressed on myeloid cells 2 (TREM2)-independent and TREM2-dependent mechanism^[33]^. A cluster of genes plays a crucial role in the TREM2-mediated processes driving the transformation of microglia from the M0 state to the DAM phenotype, as illustrated in Figure 2. Although TREM2 as a microglia receptor seems to promote the differentiation of microglia into the DAM phenotype, mutations in this receptor can influence the capacity of microglia to phagocytize the plaques linked to AD, thus worsening neuronal dysfunction. One study found that the R62H and R47H variants of TREM2 elevate the risk of AD in comparison to the more prevalent variant of TREM2^[34]^. In 2020, researchers characterized a profile of human Alzheimer’s microglia (HAM) using bulk (~100,000 microglia cells) RNA-seq on frozen postmortem brain tissue^[35]^. They identified similarities between DAM and HAM genes, but HAM exhibited accelerated aging and upregulation of APOE compared to DAM of mouse models. In addition to DAM and HAM, other microglial subsets have been discovered. Single-cell RNA sequencing of microglia in the CK-p25 mouse model has unveiled two microglia phenotypes that appear in later stages of AD^[36]^. These phenotypes are characterized by elevated expression of type-I IFN response genes such as Irf7 and Oas1a, and MHC class II genes such as Cd74 and H2-Aa. A separate study was able to discover a subset of microglia negatively associated with AD^[37]^. This subset showed increased antigen presentation of CD74 and was enriched with genes commonly lacking in AD subjects^[37]^. While evidence exists that the gut microbiome can influence the highly specific microglial differentiation processes in the hippocampus and prefrontal cortex^[38]^, the specific pathways by which the gut microbiota and their metabolites may regulate microglial differentiation remain enigmatic. As of now, a lack of knowledge in the area necessitates our reliance on conventional microglial classifications towards proinflammatory (M1) or anti-inflammatory (M2) states for the determination of mechanisms bridging the gut microbiome and AD.

## NEUROINFLAMMATION AND THE GUT-BRAIN AXIS

The gut-brain axis (GBA) is a bidirectional communication mechanism by which the human gut and brain may communicate. This is mediated directly via the vagus nerve and indirectly via multiple mechanisms that include hormonal, neuromodulatory, and immunoregulatory secretions of gut epithelial, mucosal immune, and gut microbial cells^[8]^. Due to constitutive communication between the gut and brain through the vagus nerve, it has been studied extensively in neurodegenerative diseases. Vagotomy procedures have been correlated with a lower risk of developing Parkinson’s Disease (PD)^[39]^. However, currently, there is insufficient evidence to support the efficacy of such procedures in reducing the risk of developing AD^[39]^. Instead, research has shifted focus to other GBA mediation mechanisms, such as the gut microbiome. Gut microbial communities vary and are dependent on the host individual’s establishment of a microbiome within the first few years of life^[40]^. It is well accepted that a host’s microbiome may also be modulated through many regional, diet, and lifestyle factors, as well as infection history^[30]^. When these factors confer an imbalanced gut microbial profile, associated undesirable consequences may ensue in a state termed “dysbiosis”. Dysbiosis is often accompanied by a loss of microbial diversity, an increase in proinflammatory microbes, and thus an upregulation of inflammatory markers in the central nervous system (CNS)^[41]^. Dysbiosis may also cause the breakdown of the gut epithelium, which leads to leaky gut and peripheral immunological cell exposure to bacterial components and exogenous metabolites, consequently triggering peripheral inflammation^[42]^. These effects are hypothesized to mimic the effects of mild-grade systemic sepsis, which, through cecal puncture and ligation surgery, have shown decreases in cognitive function, increased A deposition, and development of a neuroinflammatory profile^[43]^. Furthermore, the effects of the gut microbiome on peripheral inflammation are hugely evident in autoimmune diseases such as IBD^[44]^. Importantly, there are many mechanisms thoroughly reviewed by Angiulli *et al.* through which enhanced peripheral inflammation may enhance neuroinflammation and neurodegradation^[45]^. When upregulated, peripheral proinflammatory cytokines may be actively transported across the BBB and alter microglial states and activity. Moreover, when the periphery experiences a chronic proinflammatory state, oxidative and inflammatory stress may damage the integrity of the BBB, resulting in further passive diffusion of cytokines and eventual permanent alteration of the states and activity of microglia and astrocytes^[45]^. Chronic upregulation of peripheral inflammation has also been associated with reduced brain volume, particularly in hippocampal regions^[46]^. Such decreases in hippocampal volumes have been correlated to AD pathophysiology and may be mediated by microglia-mediated immunity^[47]^. It is important to note that the enhancement of peripheral inflammation by the gut microbiome does not necessitate pathogen presence or a diseased gut state. For instance, elevated peripheral inflammation, altered microglial morphology, and elevated A load have been observed in specific pathogen-free (SPF) AD-model mice, compared to germ-free (GF) AD model mice^[48]^. Moreover, fecal microbiota transplantation (FMT) of an SPF microbiome to GF mice resulted in the development of similar characteristics, although these patterns were observed in male but not female mice^[48]^. Through its intimate relation with peripheral immunity, both pathogenic and non-pathogenic, but dysbiotic, microbiomes possess numerous potential pathways through which they can interact with the brain and CNS.

Due to mounting evidence correlating gut dysbiosis, inflammation, and neurodegenerative processes, many believe that dysbiosis-induced neuroinflammation may be a primary potentiator for neurodegenerative diseases such as PD and AD^[15,41]^. Interestingly, the transfer of fecal microbiota from age-matched male mice with APPPS1 transgenes (at 21 days of age) to male transgenic mice treated with a short-term, 7-day course of antibiotics resulted in an increase in amyloidosis and the presence of microglia localized around plaques when observed at 9 weeks of age^[49]^. Moreover, antibiotic treatment and FMT have proven effective in modulating astrocyte phenotypes in manners both dependent and independent of microglia interactions^[50]^. Consistent with its ability to worsen AD pathophysiology in a deleterious context, FMT of healthy microbiomes into an AD mouse model has shown decreased Aβ plaques and NFT formation along with decreased microglial activity and cognitive impairment^[51]^. These studies have given rise to others that look to discover the specific microbes, interactions, and pathways responsible for these changes. Thus, a multitude of human clinical studies have proceeded to characterize the gut microbiome of AD patients as compared to healthy controls for the establishment of proinflammatory microbiome profiles associated with AD^[52-54]^. In a previous study, we were also able to provide supporting evidence for this hypothesis by correlating similarly elevated counts of proinflammatory bacterial species in patients with mild cognitive impairment (MCI), a precursor state to AD^[55]^.

## NEUROMODULATORY MECHANISMS WITHIN THE GUT-BRAIN-IMMUNE-AXIS

Despite the challenges in unraveling the precise molecular mechanisms through which the gut microbiome influences or is influenced by neuroinflammation, owing to the multivariate and context-dependent nature of both gut ecology and immunology, recent research is beginning to piece together the puzzle. Regulation of the immune system by the gut microbiome may occur through either metabolic pathways or direct host-microbe interactions. Direct pathways may occur through interactions between host cells and bacterial cells or components. For example, immune cells may be exposed to bacterial membranous components such as lipopolysaccharide (LPS) in the gut mucosa^[56]^. The natural metabolic processes of host microbiota also create a vast network of interplay, including the production of metabolites such as short-chain fatty acids (SCFAs) and alteration of endogenous poly-unsaturated fatty acids (PUFAs) metabolism, both of which provide indirect immunological consequences in the periphery and CNS^[19,57]^. These factors within the GBA may be actively modulated through lifestyle factors such as diet. For instance, the ethanolic extract of *Tetragonia tetragonioides* Kuntze (New Zealand spinach) has led to changes in the gut microbiome composition and insulin sensitivity, subsequently reducing hippocampal A deposition and enhancing memory^[58]^. It is through these highly modulable lifestyle factors that potential treatment methods may be available; however, this necessitates a deeper understanding of their interplay.

LPS is perhaps one of the best-characterized proinflammatory gut-brain axis mediators. LPS is a bacterial membrane secretory product of gram-negative bacteria. Gram-negative bacteria or LPS itself may be taken up by M cells or goblet cell-associated antigen pathways for transcytosis and eventual recognition in the lamina propria^[56]^. The introduction of LPS into the systemic circulation has been observed in older adults, particularly those with AD^[59]^. Somewhat more alarming, LPS has also been detected in the brains of AD patients, particularly in the hippocampus - a region highly associated with memory and learning^[60]^. A recent study revealed significantly higher levels of LPS in the temporal lobe, which were seven times greater, and in the hippocampal region, which were 21 times greater, in AD patients compared to cognitively normal control subjects^[61]^. Leaky gut caused by aging or gut dysbiosis proposes a link to LPS toxin transport across tight junctions and into the systemic circulation and the blood-brain barrier, resulting in CNS inflammation^[61]^. LPS may be recognized by TLR4 for activation of multiple pathways, including the production of NFκB^[19]^. Large amounts of peripheral circulating NFκB are known to trigger M1 microglial maturation in the brain and NFκB-mediated necroptosis^[21]^. Muhammad *et al.* demonstrated increased NFκB, TNF-α, and IL-1β levels in mouse hippocampal and microglial cell lines as a response to LPS^[62]^. Moreover, intraperitoneal injection of LPS in a mouse model was accompanied by decreases in the hippocampal expression of anti-apoptotic protein Bcl-2 and increases in the hippocampal expression of the apoptosis cascade protein caspase-3, therefore upregulating synaptic degradation and neuronal apoptosis^[62]^. A separate study demonstrated that intraperitoneal LPS administration in a mouse model also induced cognitive and motor impairments^[60]^.

SCFAs are a class of anti-inflammatory lipids that include bacterial metabolites such as acetate, propionate, and butyrate. Butyrate has a dual action in both the gut and brain. Butyrate may increase gut motility via cholinergic neurons, can be used as an energy source for epithelial cells, improves the gut blood barrier (GBB), and can improve learning and memory^[1]^. The immunomodulatory mechanisms of butyrate are vast and are widely mediated by its binding to free fatty acid receptors, which leads to the production of IL-18, a generally anti-inflammatory cytokine^[63]^. Increased peripheral butyrate as a function of a high-fiber diet is also inversely correlated to microglial production of proinflammatory cytokines such as IL-1β, TNF-α, and IL-6 in mouse models^[64]^. Acetate, upon absorption, can diffuse and cross the BBB, enabling its ability to signal satiety^[1]^. Acetate’s action in the brain also includes inhibiting NFκB pathways and decreasing neuroinflammatory markers such as IL-1 and COX-2, which are consistently observed in *in-vitro* and animal model experiments^[65]^. Like butyrate, acetate can also attenuate LPS-induced microglial activation^[64,66]^. Intriguingly, neither acetate nor butyrate improved cognitive function in healthy controls (HCs), reflecting the action of these SCFAs as neuroprotective rather than neurogenerative, thus underscoring the significance of neuroinflammation in the process of neurodegeneration^[64,66]^.

Separate from its involvement in fiber degradation and SCFA production, the gut microbiome is also capable of producing or regulating a host of other neuromodulatory and immunomodulatory compounds, such as neurotransmitters and indoles. Species of *Staphylococcus* are known to produce potent neurotransmitters such as dopamine and serotonin in the gut^[67]^. Additionally, neurotransmitters and trace amines such as γ-aminobutyric acid (GABA), acetylcholine, phenylethylamine, tyramine, and tryptamine are included within many gut microbial metabolite profiles. Local, endogenous production of many of the same compounds by enterochromaffin and enteroendocrine cells may also be altered by the activity or metabolites of the gut microbiome^[68]^. Alteration of microbially-derived histamine, catecholamines, and glutamate seem to have both direct or indirect pathways by which they may influence peripheral and CNS inflammation, therefore having possible implications in the development of AD^[69]^. Aside from inflammatory processes, evidence also shows that neurotransmission in the CNS can be strongly influenced by gut microbiota for the progression of AD pathophysiology^[70]^. Although unable to cross the BBB, many of these neuroactive metabolites act within the gut and periphery while also possessing the ability to alter CNS activity via the vagus nerve for cognition^[68]^. A clinical study by Wu *et al.* correlated disruption of tryptophan metabolism and a tryptophan-derived serotonin precursor with AD and a disrupted gut microbiome^[71]^. Highlighting the links between neuroinflammation, neurotransmitter synthesis, and the gut microbiome, animal studies have demonstrated that probiotic supplementation can enhance plasma concentration of tryptophan, thus mitigating the effects of stress-induced neuroinflammation and alterations in CNS noradrenaline content^[72,73]^. Accompanying its role in neurotransmitter synthesis, microbial tryptophan metabolism is responsible for the production of indoles - a group of tryptophan metabolites that has garnered attention within the context of both the GBA and AD for their immunomodulatory and antioxidant properties^[74]^. Compounds such as indole-3-propionate (IPA) and indoxyl-3-sulfate (I3S), both of which are exclusively produced by commensal bacteria, have been observed in CNS tissues^[75,76]^. Moreover, both compounds are aryl hydrocarbon receptor agonists that can regulate astrocyte activity, thus reducing neuroinflammation via anti-inflammatory cascades^[76]^. Their action in maintaining ROS balance, altering T-cell differentiation, and promoting an anti-inflammatory profile render their contention as a target to combat neuroinflammation in diseases such as AD^[74,77]^. While some of the major mediators are highlighted here, a great variety of understudied and unknown immune-related interactions, pathways, and metabolites that compose the greater picture of the GBA still exist.

## SPECIFIC GUT MICROBIOME SIGNATURES LINKED TO AD

**Figure 3:**
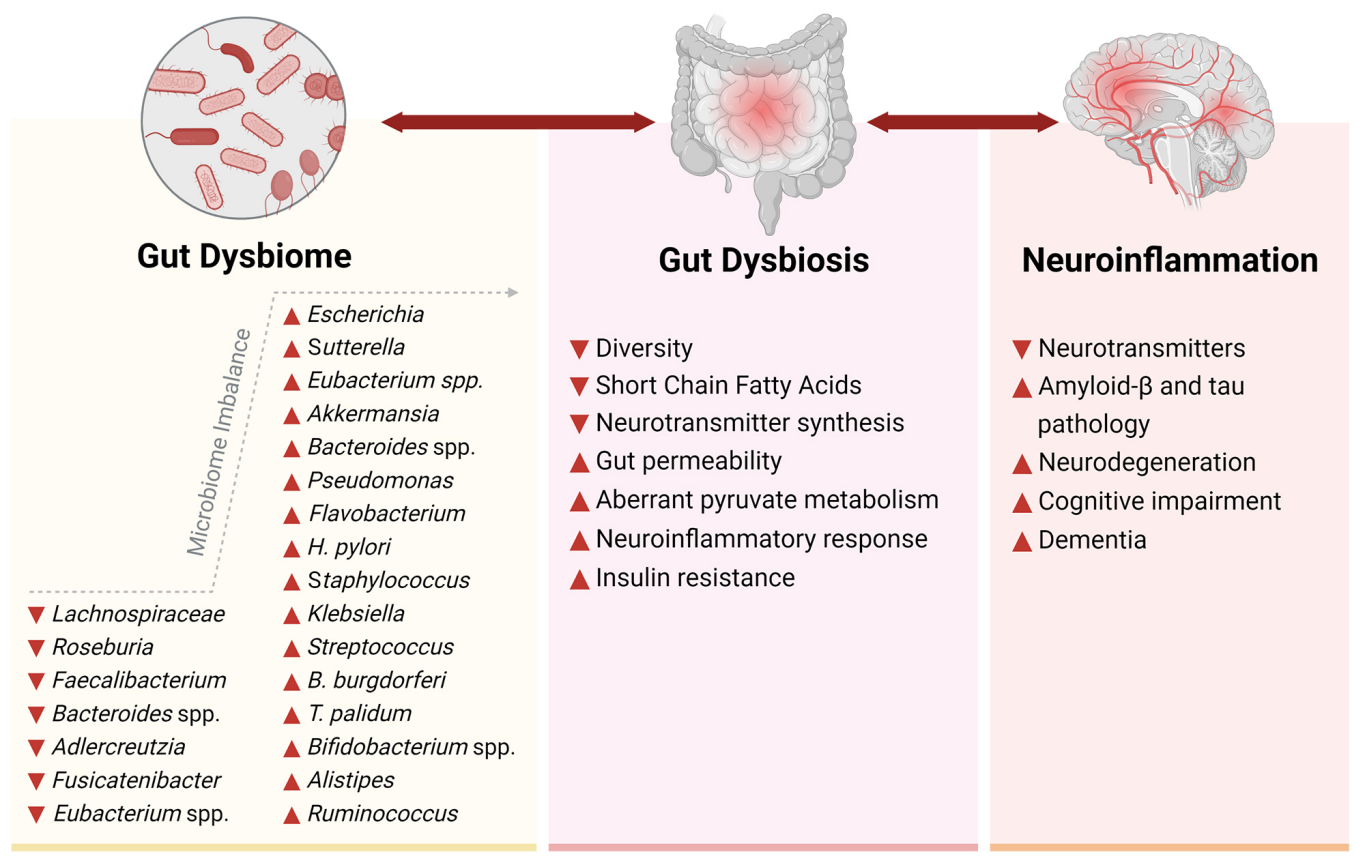
Specific bacterial signatures of gut dysbiome and pathobiome associated with neuroinflammation and AD neuropathology. The relative abundance of gut bacterial genera consistently reported as upregulated or downregulated across multiple studies comparing the gut microbiomes of AD patients with healthy controls suggests an imbalance in these gut microbial communities. This imbalance is likely connected to disruptions in gut-derived microbial metabolites, neurotransmitters, gut permeability, glucose/insulin sensitivity, and neuroinflammatory responses. Further, these imbalances instigate neuroinflammation via the gut-brain-immune axis, either via interactions between bacterial surface components and intestinal immune cells or via translocation of harmful metabolites and bacterial components across the blood-brain barrier. This progression ultimately leads to aggravated neuroinflammatory triggers and associated neuropathology observed in AD. ▲: Increased, ▼: decreased. AD: Alzheimer’s disease.

**Figure 4:**
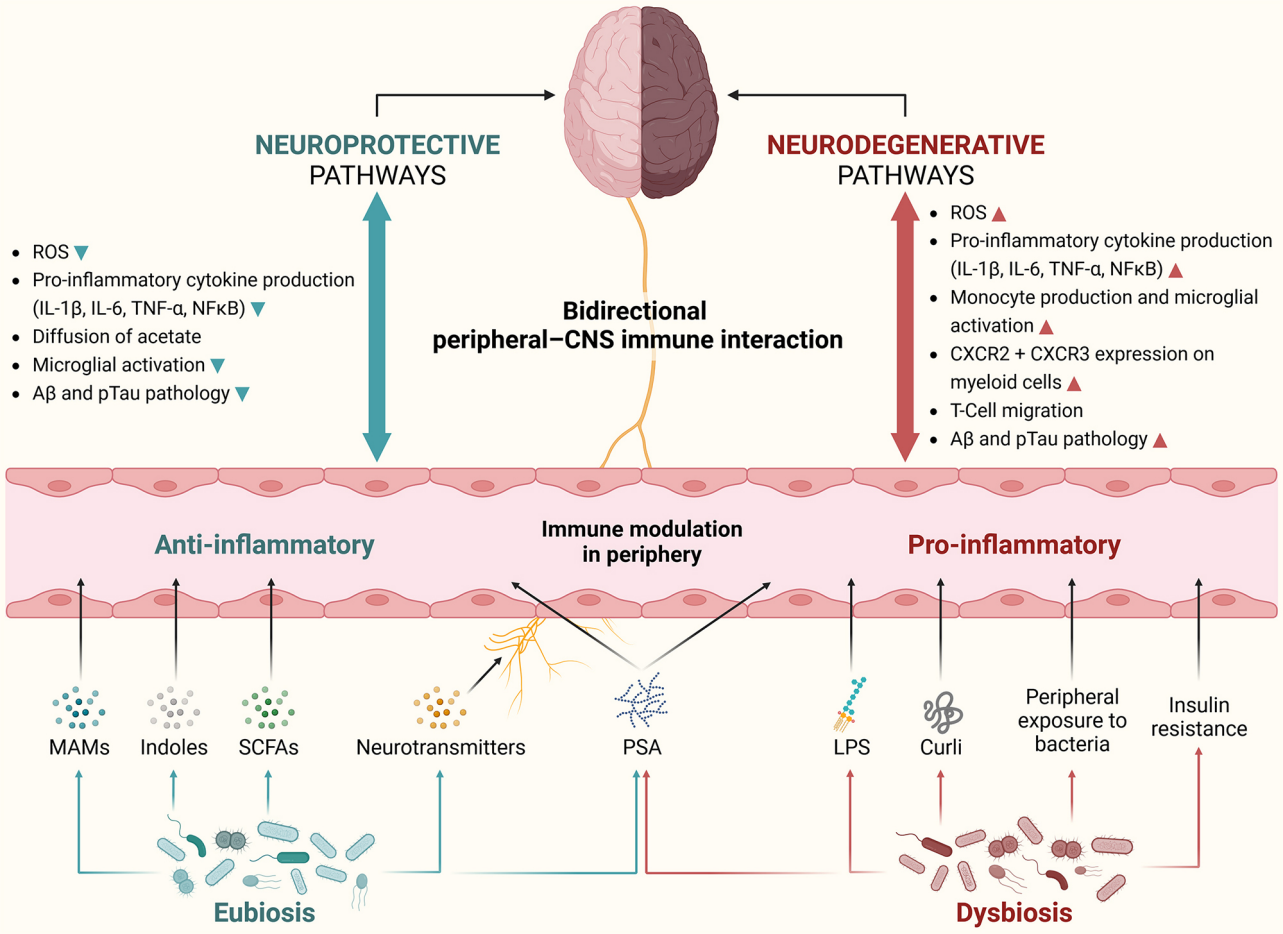
Neuroprotective and neurodegenerative pathways of microbiota-gut-brain axis describing complex bidirectional crosstalk between gut microbes, gut-derived metabolites, and the local (brain) and peripheral (blood) immune system. Gut dysbiosis initiates a proinflammatory environment in the periphery by instigating an immune response against microbial surface molecules (LPS, Curli, and PSA). This disruption adversely impacts the local blood-brain-barrier ecosystem, leading to an excessive influx of proinflammatory T-cell types, activation of M1 microglia, and an increased expression of chemokine receptors on myeloid cells. These events ultimately culminate in the accumulation of ROS and the development of Aβ and pTau pathologies associated with neurodegeneration. In contrast, a state of gut eubiosis establishes an anti-inflammatory environment in the periphery through the production of beneficial gut-derived metabolites (indoles, SCFAs, and neurotransmitters), as well as beneficial bacterial molecules (MAMs and other PSA). This fortifies the blood-brain-barrier ecosystem by facilitating the diffusion of specific metabolites such as acetate and preventing the overactivation of microglia. Ultimately, these conditions confer neuroprotective effects by reducing ROS, Aβ, and pTau pathologies. Aβ: Amyloid beta; CNS: central nervous system; CXCR: C-X-C chemokine receptors; IL: interleukins; LPS: lipopolysaccharide; MAMs: microbial anti-inflammatory molecules; NFκB: nuclear factor kappa B; PSA: polysaccharide A; pTau: phosphorylated tau protein; ROS: reactive oxygen species; SCFAs: short-chain fatty acids; TNF-α: tumor necrosis factor alpha.

Gut microbiome characterization studies that examined phylum and genus-level correlations between various progressive states of AD with non-pathogenic gut profiles and age/sex-matched HCs are reviewed. Specific gut microbiome signatures that are consistently associated with AD and MCI and their potential implications on gut dysbiosis-induced neuroinflammation are depicted in Figure 3. Obvious variations across different studies highlight previous findings that diet, region, lifestyle, and other interindividual factors are highly influential on gut microbiome composition^[30]^. Nonetheless, many region-independent AD-correlative bacteria have been discovered. The GBA operates bidirectionally, and the observational nature of these studies predisposes their inability to establish causation; however, the subsequent section explores and proposes possible causative triggers for the differential abundance and downstream mechanisms of gut microbiomes in influencing neuroinflammation and AD. A summary of the potential pathways linking the microbiota-gut-brain axis with neuroinflammatory mechanisms related to both neurodegeneration and neuroprotection is depicted in Figure 4.

### Phylum Pseudomonadota

The phylum Pseudomonadota (previously Proteobacteria) consists largely of gram-negative, LPS-bearing bacteria^[78]^. Pseudomonadota represents a collection of common resident bacteria generally present within the healthy adult microbiomes. In patients with metabolic disorders or inflammatory conditions, however, their compositional density can experience three-fold increases and may be a marker of dysbiosis^[79]^. In human clinical studies, several microbiome characterizations of AD patients and HCs showed an increased abundance of Pseudomonadota, a phylum-level taxon associated with AD^[54,80]^. Likewise, studies comparing the microbiomes of cognitively impaired patients to HCs revealed similar results^[55,81]^. While Pseudomonadota tends to generally be overrepresented in AD and MCI patients, certain studies suggest somewhat contradictory evidence as some research observed negative correlations between genera such as *Sutterella* and AD/MCI^[82,83]^ while others observed their positive correlations to AD/MCI^[52,81,84]^. Other genera such as *Escherichia/Shigella* and *Klebsiella* are consistently elevated in AD/MCI patients where differences reach significance^[54,81,82,85,86]^. The general trend of Pseudomonadota overrepresentation in AD/MCI patients may be reflective of the proinflammatory characteristics of LPS; however, inconsistencies such as those found in relative *Sutterella* abundance may be reflective of other important factors such as differential bacterial metabolic processes at the genus, species, or even strain level and thus effects on host inflammation.

Family *Enterobacteriaceae*, under the phylum Pseudomonadota, is one of the most prominent families of bacteria in most human microbiomes^[87]^. Its overrepresentation has been marked in AD patient fecal microbiomes, and one study was able to further use *Enterobacteriaceae* as a strongly differentiating biomarker for AD/MCI relative to HC microbiomes^[80]^. Moreover, *Enterobacteriaceae* exhibited progressive enrichment from HCs to MCI patients and again to AD patients^[80]^. Family *Enterobacteriaceae* and its enveloping class, *Gammaproteobacteria*, also seem to account for the marked AD-related increases in Pseudomonadota as a whole^[80]^. This may be explained by neuroinflammatory pathways, as shown by positive correlations between *Enterobacteriaceae* and brain inflammatory markers, as well as microglial activation^[88]^. One highly studied genus within *Enterobacteriaceae* is *Escherichia*, a genus of typical resident gut microbes of which certain species or strains exhibit pathogenicity^[89]^. Even in the absence of pathogenic strains, however, *Escherichia* can still be labeled as proinflammatory, thereby associated with disease^[85]^. In a classification study of AD/MCI patients and HC enterotypes, overrepresentation of *Escherichia* has also been observed to cluster with opportunistic species of *Klebsiella* and *Enterococcus*^[54]^. Overrepresentation of *Escherichia* has also been correlated to increased expression of the NLRP3 inflammasome, IL-6, IL-1β, and CXCL-2 in the blood^[85]^. Chronic excessive circulating IL-1β and IL-6 trigger the acute phase immune response and are significantly correlated with AD and depression^[90]^. CXCL-2, also referred to as macrophage inflammatory protein-2 (MIP-2), is a chemokine that is triggered through IL-1β sensing and NFκB-mediated transcription activation^[91]^. It has been linked to AD^[92]^ and acts through the chemokine receptor CXCR2 for monocyte and neutrophil recruitment, thus promoting the progression of the innate immune response^[93]^. Moreover, certain strains of *E. coli* and other bacteria are recognized for their ability to produce a peptide called curli, which resembles the Aβ_42_ peptide. Curli possesses various mechanisms through which it may be disruptive to host health, such as TLR2/1-mediated microglia and astrocyte activation for the promotion of neuroinflammation and reduction of epithelial tight junction proteins^[86]^. As demonstrated via injection of *Salmonella typhimurium*-derived curli, the protein has the ability to alter gut microbiomes and enhance proinflammatory cytokines such as IL-1, which can have downstream consequences in peripheral and CNS inflammation, therefore influencing AD pathophysiology^[94]^. Curli may also aid in protecting bacteria from host antimicrobial activities and is variably produced in a strain-dependent manner^[95]^. Moreover, CsgA is a structural subunit of curli that aggregates into curli fibers outside of the cell^[96]^. Recently, certain *E. coli*-derived CsgA homologs were shown to either accelerate or inhibit α-synuclein amyloid aggregation in the gut^[96]^. It is proposed that amyloid aggregation in the gut is then transferred to the brain via the vagus nerve, suggesting another possible mechanism between *E.coli*-produced curli protein and neurodegeneration^[96]^. This highlights that while higher order classification may be important to determine widespread functional pathways (e.g., expression of LPS across Pseudomonadota), narrow classification provides valuable insights into niche metabolic pathways that can have significant pathophysiological implications.

### Phylum Bacteroidota

Phylum Bacteroidota (formerly Bacteroidetes) represents one of the densest populations within the normal human gut microbiome^[78]^. Bacteroidota is generally composed of gram-negative bacteria and displays a vast array of phenotypes encompassing both obligate aerobic and anaerobic species and flagellated and non-motile species^[97]^. Their wide phenotypic variation has enabled their colonization of all major microbe-related regions of the gastrointestinal tract^[97]^. Owing to their high relative presence within the host gut microbiome and the extreme impact of a gut microbiome on a host, species within this phylum play an essential role in regulating host metabolism.

*Bacteroides* is one of the largest representative genera within Bacteroidota and has been extensively studied. Interestingly, the correlation of *Bacteroides* relative abundance to AD seems to be regionally dependent as many studies conducted in China correlated elevated *Bacteroides* to healthy controls^[81-83,87]^, whereas other studies conducted in the US, Spain, Austria, Netherlands, Turkey, Japan, and Thailand seem to show *Bacteroides* enrichment in AD/MCI patients^[52-54,86,98-101]^. Only a single study on Chinese cohorts found conflicting evidence and correlated *Bacteroides* enrichment with AD/MCI presence^[84]^. Similar to the extreme variability within the metabolic pathways exhibited by the enveloping phylum Bacteroidota, species within *Bacteroides* themselves exhibit high functional diversity. This may account for the observed region-dependence of AD/MCI correlations with *Bacteroides*. This is highlighted by one study in a North Carolinian cohort in which it was determined that multiple species of *Bacteroides* were significantly overrepresented in MCI, such as *B. coprocola*, *B. massiliensis*, *B. thetaiotaomicron*, and *B. xylanisolvens*, whereas the species *B. vulgatus* was correlated to HCs^[102]^. Even at the species level, however, *Bacteroides* spp. show high variability of both health promotion and decline. *B. thetaiotaomicron*, which was correlated to MCI in the study by Aljumaah *et al.*^[102]^, may exhibit proinflammatory effects such as the aberrant upregulation of IL-8 in Crohn’s disease patients^[44]^. Reflecting the context dependence of these results, however, in healthy gut microbiome profiles, *B. thetaiotaomicron* probiotic supplementation may attenuate the overproduction of IFN-γ and TNF-α and complement the growth of the butyrate producer *Faecalibacterium prausnitzii*^[103]^. Moreover, the common gut microbial species *Bacteroides fragilis* also exhibits strain-dependent pro- or anti-inflammatory properties, wherein certain strains can further be classified as enterotoxigenic^[87]^. The inflammatory impacts of *Bacteroides fragilis* are, in part, regulated by their production of the capsular polysaccharide, PSA^[104]^. One study found that PSA exposure can induce T_H_1 differentiation in primary splenic, Peyer’s patch, and mesenteric lymph node T cells for the secretion of IFN-γ, TNF-α, IL-6, and CXCL-10, as well as expression of the receptor CXCR-3^[104]^. The same study found that PSA can also induce the secretion of anti-inflammatory markers Lag3, Tim3, and PD-1^[104]^. The strain-specific characteristics of *Bacteroides*, and the context in which it is present thus play a crucial role in determining its inflammatory properties and any subsequent impacts on neuroinflammation and the development of AD pathophysiology. However, it may be more productive to shift focus towards *Bacteroides*-related gene expression rather than on the mere presence of the genus or even species when studying its correlation with AD.

### Phylum Bacillota

Bacillota is primarily composed of gram-positive bacteria with a few gram-negative exceptions, such as *Megasphaera* and *Veillonella*. Similar to Bacteroidota, Bacillota composes the other major gut-related microbe taxa, and its species exhibit a wide range of niches. Bacillota differs from Bacteroidota, however, in their enrichment in Western-style diets, whereas Bacteroidota seems to be highly enriched in individuals with high-fiber diets typical of rural African areas^[105]^. Some key Bacillota species are fiber-degrading and produce SCFAs, thus generally exhibiting anti-inflammatory effects in the gut. However, the phylum also harbors several proinflammatory species^[106]^. *Streptococcus*, for instance, has been associated with AD at the genus level independent of pathogenic species^[81,82,99]^. This may be due, in part, to certain species of *Streptococcus* that possess the ability to degrade the intraepithelial tight junctions of the GBA, therefore causing leaky gut and increasing inflammation^[107]^.

The ratio of Bacillota and Bacteroidota (labeled as the F/B ratio) in AD patients *vs.* HCs has been proposed as a possible predictive factor for AD^[87,108,109]^. This idea has been discounted in relation to other gut microbiome-related pathologies, such as obesity, as reviewed by Magne *et al.*^[110]^. This trend seems to hold true in AD/MCI-related studies wherein studies have reported directly contradictory evidence^[87,108,109]^. The differences are likely due to variations in patient medication and confounding lifestyle factors, thus subjecting the studies to interpretive bias^[110]^. It is therefore difficult to draw conclusions about AD-related Bacteroidota and Bacillota inflammation mechanisms at the phylum level.

The genera within Bacillota that seem to have the highest ameliorative impact on AD and neuroinflammation are the butyrate producers. The genus *Faecalibacterium* is a butyrate producer that has been negatively correlated to AD and MCI multiple times^[54,84,86,108,111]^. One study even found *Faecalibacterium* to be the strongest single predictive factor in the differentiation of AD patients *vs.* HCs^[111]^. The most representative *Faecalibacterium* species, *F. prausnitzii*, can cause peripheral anti-inflammatory effects via secretion of the NFκB pathway-inhibiting MAMs, and butyrate, which can upregulate T_reg_ cell differentiation for the production of anti-inflammatory cytokines IL-10 and TGF-β in colitis mouse models^[30]^. Moreover, *Faecalibacterium* has been positively correlated with amygdala and hippocampal region volume and subsequent AD, likely due, in part, to the activation of microglia^[47,86]^. This manifests as positive associations between cognitive performance scores and *Faecalibacterium*^[82]^. Contradictory evidence also exists, however, as *Faecalibacterium* overrepresentation has been reported in AD patients^[112]^. Xi *et al.* hypothesized that decreases in IFN-γ may allow for increased Aβ deposition or that strain-dependent detrimental effects may account for *Faecalibacterium* overrepresentation^[112]^. An independent study by Chen *et al.* found that *Faecalibacterium* abundance was increased in patients with AD carrying the AD risk factor *APOE4* allele compared to non-*APOE4* carriers both with and without AD^[113]^. These findings may suggest that the observed overrepresentation of *Faecalibacterium* in AD groups could be due to its confounding correlation with *APOE4*. Given the known role of *APOE4* in conferring maladaptive inflammatory and oxidative stress via mitochondrial damage, it is possible that the gene may provide a confounding variable responsible for the occasional correlation between *Faecalibacterium* and AD; however, mechanistic insights are necessary to draw further conclusions^[113,114]^. While there is evidence that it promotes a predominantly anti-inflammatory profile, regardless of direction, it is clear that *Faecalibacterium* can play a role in regulating peripheral inflammation, which is known to have downstream effects on neuroinflammation and neurodegeneration as is seen in AD.

A second primary butyrate producer is *Roseburia.* The genus *Roseburia* has been negatively correlated to AD/MCI in comparative studies with no strong contradictory evidence^[52,54,81,101,109,111]^. *Roseburia* is also a prominent butyrate producer, which confers anti-inflammatory effects on its host. The production of butyrate by *Roseburia* has been negatively correlated with proinflammatory mediators (IL-1β, IL-6, TNF-α, and IFN-γ)^[115]^. Mechanistically, the introduction of *Roseburia hominis* to the microbiomes of GF rats indicated decreased microglial activation and IFN-γ production as a function of butyrate production. *Roseburia* treatment also alleviated the GF rats’ depressive behaviors associated with AD^[116]^. Interestingly, certain *Roseburia* species are implicated in the metabolism of phenylalanine (Phe) in the gut. One study discovered 13 different Phe-metabolizing enzymes produced by *Roseburia* while noting that the genus accounted for the largest proportion of Phe-derived metabolites as compared to related taxa. As Phe can induce T_H_1 cell differentiation *in vitro* and in mouse models, its role in inflammation and neuroinflammation is of note and supports the hypothesis that *Roseburia* may influence inflammation via Phe metabolic pathways^[117]^. In phenylketonuria patients, Phe accumulation is known to impair brain synapses^[118]^. Phe accumulation and dysregulated hippocampal Phe metabolism have also been separately associated with AD pathophysiology^[119]^. Moreover, transgenic AD-model mice show significantly increased blood and stool concentrations of Phe^[117]^. Evidence, therefore, points to *Roseburia* as a bridge between increased Phe concentrations and neuroinflammation in AD, although more research is required for further insights into this speculation.

### Phylum Verrucomicrobiota

Verrucomicrobiota is a phylum of bacteria that includes many gut commensals. While analysis at the phylum level lacks evidence for correlation to AD, the genus *Akkermansia* has been repeatedly correlated to AD^[54,100,111]^. Within the genus, the highly represented species, *A. muciniphila*, has been positively correlated to medial temporal atrophy^[82]^. As *A. muciniphila* has been studied for probiotic benefits, these correlations to AD raise alarm. The species is gram-negative and is known for its ability to metabolize mucin - its primary energy source - and produce the SCFAs acetate and propionate^[30]^. Counterintuitively, however, *A. muciniphila* has generally proinflammatory effects on the host as it has been linked to increases in IFN-γ and TNF-α and decreases in IL-10 and IL-4^[30]^. It has been proposed that, in elderly patients, mucin degradation may lead to increased epithelial permeability and thus worsened AD through leaky gut^[84]^. Increases in *A. muciniphila* may, however, also be a mere residual effect of other AD-related pathophysiological markers. The cytokine IL-33 is linked to AD and promotes T_H_2 cell differentiation for the secretion of IL-13 and goblet cell differentiation^[120]^. The overexpression of IL-33 represents an anti-inflammatory pathway and seems to serve as a rescue mechanism, as cognitive impairment in AD may be preserved by its action^[120]^. *B. thetaiotaomicron*, previously correlated to MCI^[102]^, is also evidenced to increase goblet cell differentiation^[121]^. As goblet cells are the human gut’s primary mucin producers, their increase due to AD-related markers may render the role of paralleled increases in *A. muciniphila* as a mere bystander effect. Therefore, it is crucial to conduct future mechanistic studies to elucidate the interconnected mechanisms involving IL-33, goblet cell differentiation, and their impact on the abundance of *Akkermansia* in relation to the pathophysiology of AD.

### Infectious microbes and pathobionts

As gut microbiome characterization studies focusing on AD and MCI often exclude individuals with gut pathologies such as IBD or infections, the potential impact of specific gut pathogens on neurodegeneration has largely remained underexplored with few noted exceptions. The species *Helicobacter pylori* is a known pathogen of the phylum Pseudomonadota that causes peptic ulcers. Its presence has been strongly correlated to AD^[122]^ and its eradication after infection has been correlated to decreased risk for AD progression^[123]^. The current proposed hypotheses bridging chronic *H. pylori* infection and AD posits that *H. pylori* can either cross the BBB while lying dormant in monocytes or enter the brain via the oral-nasal-olfactory systems wherein it may provoke damaging proinflammatory events^[22]^. Certain infectious species within the phylum Spirochaetota, such as *Borrelia burgdorferi* and *Treponema pallidum*, the causative bacteria of Lyme disease and Syphilis, respectively, have also been correlated to AD^[124]^. *B. burgdorferi* can form biofilms in the brain using quorum sensing mechanisms and bacterial amyloids, specifically curli fibers. The curli peptides trigger TLR-2 for the upregulation of NFκB and TNF-α, which may trigger neuronal death^[125]^. *T. pallidum*, an even stronger neuroinflammatory microbe, is well known to colonize and persist in the brain. Syphilis thus often presents with neuroinflammation and dementia within the years following a primary infection event^[126]^. The known causative roles that infectious microbes play in neuroinflammation, and neurodegenerative pathways highlight and support the suggested role of proinflammatory gut commensals in conferring negative neurological health effects when chronically overrepresented. This highlights an additional opportunity to investigate the neuromodulatory mechanisms induced by the pathobiome, specifically focusing on the influence of specific pathogenic clades within the gut microbiome, as these clades are frequently overlooked in sequencing workflows due to their underrepresentation.

## CONCLUSIONS AND PROSPECTS

Current treatment strategies aimed at targeting AD biomarkers (Aβ and pTau) have thus far been ineffective against successful amelioration of neurodegeneration. Instead, a targeted approach to modulating neuroinflammatory processes through the ever-present gut microbiome is under intense research. The neurodegenerative effects of gut-derived inflammatory processes seem to be largely mediated by the M1 microglia of the innate immune system, and M1-stimulating T_H_1 and T_H_17 cell infiltration of the BBB. The absorption of bacterial metabolites and recognition of their membranous components can affect neuroinflammation through the potentiation of complex signaling cascades assembling into proinflammatory milieus in the periphery. The current attempts to establish associations between phyla and neuroinflammation related to AD have yielded limited success, primarily due to the considerable variations in gut ecological niches and metabolic pathways. However, at the genus level, there have been promising associations between specific genera and AD-related neuroinflammation. For instance, *Roseburia* and *Escherichia* have been indisputably negatively and positively correlated to AD, respectively. Nevertheless, to gain a comprehensive understanding of the potential benign or harmful responses of ambiguous species such as *Bacteroides fragilis*, *Escherichia coli*, and *Faecalibacterium prausnitzii*, a strain-level discriminatory approach is required to understand their distinct roles in AD progression. Additionally, conducting mechanistic studies using monocultures of gut pathogens and fecal transplantations from AD patients into GF or re-conventionalized SPF rodents, coupled with the integrated systems approach, would be instrumental in unraveling the crucial host-microbe interactions that contribute to the inflammatory aspects of neurodegeneration.

## DECLARATIONS

### Copyright

© The Author(s) 2023.

## References

[1] Sochocka M, Donskow-Łysoniewska K, Diniz BS, Kurpas D, Brzozowska E, Leszek J (2019). The gut microbiome alterations and inflammation-driven pathogenesis of Alzheimer’s disease - a critical review. Mol Neurobiol.

[2] Alam R, Abdolmaleky HM, Zhou JR (2017). Microbiome, inflammation, epigenetic alterations, and mental diseases. Am J Med Genet B Neuropsychiatr Genet.

[3] Askarova S, Umbayev B, Masoud AR (2020). The links between the gut microbiome, aging, modern lifestyle and Alzheimer’s disease. Front Cell Infect Microbiol.

[4] Santiago JA, Potashkin JA (2021). The impact of disease comorbidities in Alzheimer’s disease. Front Aging Neurosci.

[5] Leng F, Hinz R, Gentleman S (2023). Neuroinflammation is independently associated with brain network dysfunction in Alzheimer’s disease. Mol Psychiatry.

[6] Griciuc A, Serrano-Pozo A, Parrado AR (2013). Alzheimer’s disease risk gene *CD33* inhibits microglial uptake of amyloid beta. Neuron.

[7] Leng F, Edison P (2021). Neuroinflammation and microglial activation in Alzheimer disease: where do we go from here?. Nat Rev Neurol.

[8] Dai CL, Liu F, Iqbal K, Gong CX (2022). Gut microbiota and immunotherapy for Alzheimer’s disease. Int J Mol Sci.

[9] Bhattacharjee S, Lukiw WJ (2013). Alzheimer’s disease and the microbiome. Front Cell Neurosci.

[10] https://www.who.int/news-room/fact-sheets/detail/dementia.

[11] https://www.alzint.org/resource/world-alzheimer-report-2022/.

[12] Frisoni GB, Altomare D, Thal DR (2022). The probabilistic model of Alzheimer disease: the amyloid hypothesis revised. Nat Rev Neurosci.

[13] Hoe HS, Lee HK, Pak DT (2012). The upside of APP at synapses. CNS Neurosci Ther.

[14] Duka V, Lee JH, Credle J (2013). Identification of the sites of tau hyperphosphorylation and activation of tau kinases in synucleinopathies and Alzheimer’s diseases. PLoS One.

[15] Lin L, Zheng LJ, Zhang LJ (2018). Neuroinflammation, gut microbiome, and Alzheimer’s disease. Mol Neurobiol.

[16] Yang Y, Guo L, Wang J (2022). Arglabin regulates microglia polarization to relieve neuroinflammation in Alzheimer’s disease. J Biochem Mol Toxicol.

[17] Zhang Y, Niu C (2022). Relation of CDC42, Th1, Th2, and Th17 cells with cognitive function decline in Alzheimer’s disease. Ann Clin Transl Neurol.

[18] Halle A, Hornung V, Petzold GC (2008). The NALP3 inflammasome is involved in the innate immune response to amyloid-β. Nat Immunol.

[19] Thakur S, Dhapola R, Sarma P, Medhi B, Reddy DH (2023). Neuroinflammation in Alzheimer’s disease: current progress in molecular signaling and therapeutics. Inflammation.

[20] Dubenko OE, Chyniak OS, Potapov OO (2021). Levels of proinflammatory cytokines IL-17 and IL-23 in patients with Alzheimer’s disease, mild cognitive impairment and vascular dementia. Wiad Lek.

[21] Dong Y, Yu H, Li X (2022). Hyperphosphorylated tau mediates neuronal death by inducing necroptosis and inflammation in Alzheimer’s disease. J Neuroinflammation.

[22] Doulberis M, Kotronis G, Thomann R (2018). Review: Impact of *Helicobacter pylori* on Alzheimer’s disease: what do we know so far?. Helicobacter.

[23] Koenigsknecht-Talboo J, Landreth GE (2005). Microglial phagocytosis induced by fibrillar beta-amyloid and IgGs are differentially regulated by proinflammatory cytokines. J Neurosci.

[24] Ganguly U, Kaur U, Chakrabarti SS (2021). Oxidative stress, neuroinflammation, and NADPH oxidase: implications in the pathogenesis and treatment of Alzheimer’s disease. Oxid Med Cell Longev.

[25] Browne TC, McQuillan K, McManus RM, O’Reilly JA, Mills KHG, Lynch MA (2013). IFN-γ production by amyloid β-specific Th1 cells promotes microglial activation and increases plaque burden in a mouse model of Alzheimer’s disease. J Immunol.

[26] Westermann J, Pabst R (1996). How organ-specific is the migration of 'naive' and 'memory' T cells?. Immunol Today.

[27] Satarkar D, Patra C (2022). Evolution, expression and functional analysis of CXCR3 in neuronal and cardiovascular diseases: a narrative review. Front Cell Dev Biol.

[28] Ye X, Chen J, Pan J (2023). Interleukin-17 promotes the infiltration of CD8+ T cells into the brain in a mouse model for Alzheimer’s disease. Immunol Invest.

[29] Cristiano C, Volpicelli F, Lippiello P (2019). Neutralization of IL-17 rescues amyloid-β-induced neuroinflammation and memory impairment. Br J Pharmacol.

[30] Effendi RMRA, Anshory M, Kalim H (2022). *Akkermansia muciniphila* and *Faecalibacterium prausnitzii* in immune-related diseases. Microorganisms.

[31] Masuda T, Sankowski R, Staszewski O, Prinz M (2020). Microglia heterogeneity in the single-cell era. Cell Rep.

[32] Kubick N, Henckell Flournoy PC, Klimovich P, Manda G, Mickael ME (2020). What has single-cell RNA sequencing revealed about microglial neuroimmunology?. Immun Inflamm Dis.

[33] Keren-Shaul H, Spinrad A, Weiner A (2017). A unique microglia type associated with restricting development of Alzheimer’s disease. Cell.

[34] Zhou Y, Song WM, Andhey PS (2020). Human and mouse single-nucleus transcriptomics reveal TREM2-dependent and TREM2-independent cellular responses in Alzheimer’s disease. Nat Med.

[35] Srinivasan K, Friedman BA, Etxeberria A (2020). Alzheimer’s patient microglia exhibit enhanced aging and unique transcriptional activation. Cell Rep.

[36] Mathys H, Adaikkan C, Gao F (2017). Temporal tracking of microglia activation in neurodegeneration at single-cell resolution. Cell Rep.

[37] Olah M, Menon V, Habib N (2020). Single cell RNA sequencing of human microglia uncovers a subset associated with Alzheimer’s disease. Nat Commun.

[38] Huang Y, Wu J, Zhang H (2023). The gut microbiome modulates the transformation of microglial subtypes. Mol Psychiatry.

[39] Nguyen VTT, Endres K (2021). A crate of Pandora: do amyloids from bacteria promote Alzheimer’s disease?. Neural Regen Res.

[40] Xiao L, Wang J, Zheng J, Li X, Zhao F (2021). Deterministic transition of enterotypes shapes the infant gut microbiome at an early age. Genome Biol.

[41] Bonsack B, Jiang RH, Borlongan CV (2020). A gut feeling about stroke reveals gut-brain axis’ active role in homeostasis and dysbiosis. J Cereb Blood Flow Metab.

[42] Seguella L, Sarnelli G, Esposito G (2020). Leaky gut, dysbiosis, and enteric glia activation: the trilogy behind the intestinal origin of Parkinson’s disease. Neural Regen Res.

[43] Giridharan VV, Catumbela CSG, Catalão CHR

[44] Edwards LA, Lucas M, Edwards EA (2011). Aberrant response to commensal *Bacteroides thetaiotaomicron* in Crohn’s disease: an ex vivo human organ culture study. Inflamm Bowel Dis.

[45] Angiulli F, Conti E, Zoia CP (2021). Blood-based biomarkers of neuroinflammation in Alzheimer’s disease: a central role for periphery?. Diagnostics.

[46] Walker KA, Hoogeveen RC, Folsom AR (2017). Midlife systemic inflammatory markers are associated with late-life brain volume: the ARIC study. Neurology.

[47] Lancaster TM, Hill MJ, Sims R, Williams J (2019). Microglia - mediated immunity partly contributes to the genetic association between Alzheimer’s disease and hippocampal volume. Brain Behav Immun.

[48] Dodiya HB, Kuntz T, Shaik SM (2019). Sex-specific effects of microbiome perturbations on cerebral Aβ amyloidosis and microglia phenotypes. J Exp Med.

[49] Dodiya HB, Lutz HL, Weigle IQ (2022). Gut microbiota-driven brain Aβ amyloidosis in mice requires microglia. J Exp Med.

[50] Chandra S, Di Meco A, Dodiya HB

[51] Kim MS, Kim Y, Choi H (2020). Transfer of a healthy microbiota reduces amyloid and tau pathology in an Alzheimer’s disease animal model. Gut.

[52] Cammann D, Lu Y, Cummings MJ (2023). Genetic correlations between Alzheimer’s disease and gut microbiome genera. Sci Rep.

[53] Vogt NM, Kerby RL, Dill-McFarland KA (2017). Gut microbiome alterations in Alzheimer’s disease. Sci Rep.

[54] Yıldırım S, Nalbantoğlu ÖU, Bayraktar A (2022). stratification of the gut microbiota composition landscape across the Alzheimer’s disease continuum in a turkish cohort. mSystems.

[55] Nagpal R, Neth BJ, Wang S, Craft S, Yadav H (2019). Modified Mediterranean-ketogenic diet modulates gut microbiome and short-chain fatty acids in association with Alzheimer’s disease markers in subjects with mild cognitive impairment. EBioMedicine.

[56] Hollander D, Kaunitz JD (2020). The “Leaky Gut”: tight junctions but loose associations?. Dig Dis Sci.

[57] Chen C, Liao J, Xia Y (2022). Gut microbiota regulate Alzheimer’s disease pathologies and cognitive disorders via PUFA-associated neuroinflammation. Gut.

[58] Kim DS, Ko BS, Ryuk JA, Park S (2020). Tetragonia tetragonioides protected against memory dysfunction by elevating hippocampal amyloid-β deposition through potentiating insulin signaling and altering gut microbiome composition. Int J Mol Sci.

[59] Zhan X, Stamova B, Sharp FR (2018). Lipopolysaccharide associates with amyloid plaques, neurons and oligodendrocytes in Alzheimer’s disease brain: a review. Front Aging Neurosci.

[60] Zhao Y, Jaber V, Lukiw WJ (2017). Secretory products of the human GI tract microbiome and their potential impact on Alzheimer’s disease (AD): detection of lipopolysaccharide (LPS) in AD hippocampus. Front Cell Infect Microbiol.

[61] Zhao Y, Cong L, Jaber V, Lukiw WJ (2017). Microbiome-derived lipopolysaccharide enriched in the perinuclear region of Alzheimer’s disease brain. Front Immunol.

[62] Muhammad T, Ikram M, Ullah R, Rehman SU, Kim MO (2019). Hesperetin, a citrus flavonoid, attenuates LPS-induced neuroinflammation, apoptosis and memory impairments by modulating TLR4/NF-κB signaling. Nutrients.

[63] Salvi PS, Cowles RA (2021). Butyrate and the intestinal epithelium: modulation of proliferation and inflammation in homeostasis and disease. Cells.

[64] Matt SM, Allen JM, Lawson MA, Mailing LJ, Woods JA, Johnson RW (2018). Butyrate and dietary soluble fiber improve neuroinflammation associated with aging in mice. Front Immunol.

[65] Liu J, Li H, Gong T (2020). Anti-neuroinflammatory effect of short-chain fatty acid acetate against Alzheimer’s disease via upregulating GPR41 and inhibiting ERK/JNK/NF-κB. J Agric Food Chem.

[66] Reisenauer CJ, Bhatt DP, Mitteness DJ (2011). Acetate supplementation attenuates lipopolysaccharide-induced neuroinflammation. J Neurochem.

[67] Luqman A, Nega M, Nguyen MT, Ebner P, Götz F (2018). SadA-expressing staphylococci in the human gut show increased cell adherence and internalization. Cell Rep.

[68] Chen Y, Xu J, Chen Y (2021). Regulation of neurotransmitters by the gut microbiota and effects on cognition in neurological disorders. Nutrients.

[69] Qian XH, Song XX, Liu XL, Chen SD, Tang HD (2021). Inflammatory pathways in Alzheimer’s disease mediated by gut microbiota. Ageing Res Rev.

[70] Goyal D, Ali SA, Singh RK (2021). Emerging role of gut microbiota in modulation of neuroinflammation and neurodegeneration with emphasis on Alzheimer’s disease. Prog Neuropsychopharmacol Biol Psychiatry.

[71] Wu L, Han Y, Zheng Z (2021). Altered gut microbial metabolites in amnestic mild cognitive impairment and Alzheimer’s disease: signals in host-microbe interplay. Nutrients.

[72] Desbonnet L, Garrett L, Clarke G, Kiely B, Cryan JF, Dinan TG (2010). Effects of the probiotic Bifidobacterium infantis in the maternal separation model of depression. Neuroscience.

[73] Desbonnet L, Garrett L, Clarke G, Bienenstock J, Dinan TG (2008). The probiotic Bifidobacteria infantis: an assessment of potential antidepressant properties in the rat. J Psychiatr Res.

[74] Pappolla MA, Perry G, Fang X, Zagorski M, Sambamurti K, Poeggeler B (2021). Indoles as essential mediators in the gut-brain axis. Their role in Alzheimer’s disease. Neurobiol Dis.

[75] Chyan YJ, Poeggeler B, Omar RA (1999). Potent neuroprotective properties against the Alzheimer beta-amyloid by an endogenous melatonin-related indole structure, indole-3-propionic acid. J Biol Chem.

[76] Rothhammer V, Mascanfroni ID, Bunse L (2016). Type I interferons and microbial metabolites of tryptophan modulate astrocyte activity and central nervous system inflammation via the aryl hydrocarbon receptor. Nat Med.

[77] Lin J, Sun-Waterhouse D, Cui C (2022). The therapeutic potential of diet on immune-related diseases: based on the regulation on tryptophan metabolism. Crit Rev Food Sci Nutr.

[78] Oren A, Garrity GM (2021). Valid publication of the names of forty-two phyla of prokaryotes. Int J Syst Evol Microbiol.

[79] Shin NR, Whon TW, Bae JW (2015). Proteobacteria: microbial signature of dysbiosis in gut microbiota. Trends Biotechnol.

[80] Liu P, Wu L, Peng G (2019). Altered microbiomes distinguish Alzheimer’s disease from amnestic mild cognitive impairment and health in a Chinese cohort. Brain Behav Immun.

[81] Lu S, Yang Y, Xu Q (2022). Gut microbiota and targeted biomarkers analysis in patients with cognitive impairment. Front Neurol.

[82] Li B, He Y, Ma J (2019). Mild cognitive impairment has similar alterations as Alzheimer’s disease in gut microbiota. Alzheimers Dement.

[83] Zhou Y, Wang Y, Quan M, Zhao H, Jia J (2021). Gut microbiota changes and their correlation with cognitive and neuropsychiatric symptoms in Alzheimer’s disease. J Alzheimers Dis.

[84] Wang Y, Li L, Zhao X (2022). Intestinal microflora changes in patients with mild Alzheimer’s disease in a Chinese cohort. J Alzheimers Dis.

[85] Cattaneo A, Cattane N, Galluzzi S, INDIA-FBP Group (2017). Association of brain amyloidosis with pro-inflammatory gut bacterial taxa and peripheral inflammation markers in cognitively impaired elderly. Neurobiol Aging.

[86] Wanapaisan P, Chuansangeam M, Nopnipa S (2022). Association between gut microbiota with mild cognitive impairment and Alzheimer’s disease in a Thai population. Neurodegener Dis.

[87] Zhuang ZQ, Shen LL, Li WW (2018). Gut microbiota is altered in patients with Alzheimer’s disease. J Alzheimers Dis.

[88] Kang DJ, Betrapally NS, Ghosh SA (2016). Gut microbiota drive the development of neuroinflammatory response in cirrhosis in mice. Hepatology.

[89] Xia Y, Wang J, Fang X, Dou T, Han L, Yang C (2021). Combined analysis of metagenomic data revealed consistent changes of gut microbiome structure and function in inflammatory bowel disease. J Appl Microbiol.

[90] Ng A, Tam WW, Zhang MW (2018). IL-1β, IL-6, TNF- α and CRP in elderly patients with depression or Alzheimer’s disease: systematic review and meta-analysis. Sci Rep.

[91] Burke SJ, Lu D, Sparer TE (2014). NF-κB and STAT1 control CXCL1 and CXCL2 gene transcription. Am J Physiol Endocrinol Metab.

[92] Bryant AG, Hu M, Carlyle BC (2020). Cerebrovascular senescence is associated with tau pathology in Alzheimer’s disease. Front Neurol.

[93] Vallès A, Grijpink-Ongering L, de Bree FM, Tuinstra T, Ronken E (2006). Differential regulation of the CXCR2 chemokine network in rat brain trauma: implications for neuroimmune interactions and neuronal survival. Neurobiol Dis.

[94] Bessho S, Grando KCM, Kyrylchuk K (2023). Systemic exposure to bacterial amyloid curli alters the gut mucosal immune response and the microbiome, exacerbating *Salmonella*-induced arthritis. Gut Microbes.

[95] Biesecker SG, Nicastro LK, Wilson RP, Tükel Ç (2018). The functional amyloid curli protects escherichia coli against complement-mediated bactericidal activity. Biomolecules.

[96] Bhoite SS, Han Y, Ruotolo BT, Chapman MR (2022). Mechanistic insights into accelerated α-synuclein aggregation mediated by human microbiome-associated functional amyloids. J Biol Chem.

[97] Thomas F, Hehemann JH, Rebuffet E, Czjzek M, Michel G (2011). Environmental and gut bacteroidetes: the food connection. Front Microbiol.

[98] Saji N, Murotani K, Hisada T (2019). The relationship between the gut microbiome and mild cognitive impairment in patients without dementia: a cross-sectional study conducted in Japan. Sci Rep.

[99] Stadlbauer V, Engertsberger L, Komarova I (2020). Dysbiosis, gut barrier dysfunction and inflammation in dementia: a pilot study. BMC Geriatr.

[100] Ubeda C, Vázquez-Carretero MD, Luque-Tirado A (2022). Fecal volatile organic compounds and microbiota associated with the progression of cognitive impairment in Alzheimer’s disease. Int J Mol Sci.

[101] Verhaar BJH, Hendriksen HMA, de Leeuw FA (2022). Gut microbiota composition is related to AD pathology. Front Immunol.

[102] Aljumaah MR, Bhatia U, Roach J, Gunstad J, Azcarate Peril MA (2022). The gut microbiome, mild cognitive impairment, and probiotics: a randomized clinical trial in middle-aged and older adults. Clin Nutr.

[103] Sitkin S, Pokrotnieks J (2019). Clinical potential of anti-inflammatory effects of faecalibacterium prausnitzii and butyrate in inflammatory bowel disease. Inflamm Bowel Dis.

[104] Alvarez CA, Jones MB, Hambor J, Cobb BA (2020). Characterization of polysaccharide A response reveals interferon responsive gene signature and immunomodulatory marker expression. Front Immunol.

[105] De Filippo C, Cavalieri D, Di Paola M (2010). Impact of diet in shaping gut microbiota revealed by a comparative study in children from Europe and rural Africa. Proc Natl Acad Sci U S A.

[106] Sun Y, Zhang S, Nie Q (2022). Gut firmicutes: relationship with dietary fiber and role in host homeostasis. Crit Rev Food Sci Nutr.

[107] Soriano M, Santi I, Taddei A, Rappuoli R, Grandi G, Telford JL (2006). Group B Streptococcus crosses human epithelial cells by a paracellular route. J Infect Dis.

[108] Duan M, Liu F, Fu H, Lu S, Wang T (2021). Preoperative microbiomes and intestinal barrier function can differentiate prodromal Alzheimer’s disease from normal neurocognition in elderly patients scheduled to undergo orthopedic surgery. Front Cell Infect Microbiol.

[109] Sheng C, Lin L, Lin H, Wang X, Han Y, Liu SL (2021). Altered gut microbiota in adults with subjective cognitive decline: the SILCODE study. J Alzheimers Dis.

[110] Magne F, Gotteland M, Gauthier L (2020). The firmicutes/bacteroidetes ratio: a relevant marker of gut dysbiosis in obese patients?. Nutrients.

[111] Ling Z, Zhu M, Yan X (2020). Structural and functional dysbiosis of fecal microbiota in Chinese patients with Alzheimer’s disease. Front Cell Dev Biol.

[112] Xi J, Ding D, Zhu H (2021). Disturbed microbial ecology in Alzheimer’s disease: evidence from the gut microbiota and fecal metabolome. BMC Microbiol.

[113] Chen XX, Zeng MX, Cai D, Zhou HH, Wang YJ, Liu Z (2023). Correlation between APOE4 gene and gut microbiota in Alzheimer’s disease. Benef Microbes.

[114] Botchway BO, Okoye FC, Chen Y, Arthur WE, Fang M (2022). Alzheimer disease: recent updates on apolipoprotein E and gut microbiome mediation of oxidative stress, and prospective interventional agents. Aging Dis.

[115] Stražar M, Mourits VP, Koeken VACM (2021). The influence of the gut microbiome on BCG-induced trained immunity. Genome Biol.

[116] Song L, Sun Q, Zheng H (2022). Roseburia hominis alleviates neuroinflammation via short-chain fatty acids through histone deacetylase inhibition. Mol Nutr Food Res.

[117] Wang X, Sun G, Feng T (2019). Sodium oligomannate therapeutically remodels gut microbiota and suppresses gut bacterial amino acids-shaped neuroinflammation to inhibit Alzheimer’s disease progression. Cell Res.

[118] Martynyuk AE, Glushakov AV, Sumners C, Laipis PJ, Dennis DM, Seubert CN (2005). Impaired glutamatergic synaptic transmission in the PKU brain. Mol Genet Metab.

[119] Liu P, Yang Q, Yu N (2021). Phenylalanine metabolism is dysregulated in human hippocampus with Alzheimer’s disease related pathological changes. J Alzheimers Dis.

[120] Rao X, Hua F, Zhang L (2022). Dual roles of interleukin-33 in cognitive function by regulating central nervous system inflammation. J Transl Med.

[121] Wrzosek L, Miquel S, Noordine ML (2013). *Bacteroides thetaiotaomicron* and *Faecalibacterium prausnitzii* influence the production of mucus glycans and the development of goblet cells in the colonic epithelium of a gnotobiotic model rodent. BMC Biol.

[122] Kountouras J, Tsolaki M, Gavalas E (2006). Relationship between Helicobacter pylori infection and Alzheimer disease. Neurology.

[123] Chang YP, Chiu GF, Kuo FC (2013). Eradication of *Helicobacter pylori* is associated with the progression of dementia: a population-based study. Gastroenterol Res Pract.

[124] Holmes C, Cotterell D (2009). Role of infection in the pathogenesis of Alzheimer’s disease: implications for treatment. CNS Drugs.

[125] Allen HB (2016). Alzheimer’s disease: assessing the role of spirochetes, biofilms, the immune system, and amyloid-β with regard to potential treatment and prevention. J Alzheimers Dis.

[126] Miklossy J (2015). Historic evidence to support a causal relationship between spirochetal infections and Alzheimer’s disease. Front Aging Neurosci.

